# Location-Specific Orientation Set Is Independent of the Horizontal Benefit with or Without Object Boundaries

**DOI:** 10.3390/vision3020030

**Published:** 2019-06-14

**Authors:** Zhe Chen, Ailsa Humphries, Kyle R. Cave

**Affiliations:** 1 Department of Psychology, University of Canterbury, Christchurch 8140, New Zealand; 2 Department of Psychological and Brain Sciences, University of Massachusetts, Amherst, MA 01003, USA

**Keywords:** attentional set, orientation, horizontal benefit, object-based attention, location cuing

## Abstract

Chen and Cave (2019) showed that facilitation in visual comparison tasks that had previously been attributed to object-based attention could more directly be explained as facilitation in comparing two shapes that are configured horizontally rather than vertically. They also cued the orientation of the upcoming stimulus configuration without cuing its location and found an asymmetry: the orientation cue only enhanced performance for vertical configurations. The current study replicates the horizontal benefit in visual comparison and again demonstrates that it is independent of surrounding object boundaries. In these experiments, the cue is informative about the location of the target configuration as well as its orientation, and it enhances performance for both horizontal and vertical configurations; there is no asymmetry. Either a long or a short cue can enhance performance when it is valid. Thus, Chen and Cave’s cuing asymmetry seems to reflect unusual aspects of an attentional set for orientation that must be established without knowing the upcoming stimulus location. Taken together, these studies show that a location-specific cue enhances comparison independently of the horizontal advantage, while a location-nonspecific cue produces a different type of attentional set that does not enhance comparison in horizontal configurations.

## 1. Introduction

The importance of perceptual group or object in guiding visual attention has long been recognized [1,2,3,4,5,6]. Francolini and Egeth [2] used Stroop-like stimuli [7] with color as the target defining feature, and found increased reaction times (RTs) when the inconsistent information had the feature of the to-be-attended target items, but not when it had the feature of the to-be-ignored distractor items. Harms and Bundesen [3] investigated the effect of color similarity between a target and distractors when the target was defined by spatial location. Performance was impaired more when distractors had the same color as the target even though color was task irrelevant. Using a memory recall task, Kahneman and Henik [5] briefly presented stimuli to participants and found that they tended to recall adjacent items together if these items belonged to the same perceptual group, but not if they belonged to different perceptual groups. Duncan [1] explored the number of objects that could be attended to simultaneously without a cost. Participants saw two superimposed objects and reported either one feature or two features that belonged to the same object or to different objects. Reporting a second feature incurred a cost only when the two features belonged to different objects. These and other related findings demonstrated the importance of perceptual group or object in visual information processing, and they led to the proposal that attention is guided by perceptual object organization in addition to spatial location [4,5]. 

Evidence for object-based attention has been demonstrated in a number of experimental paradigms [8]. The most often used is a modification of the spatial cuing paradigm [9,10]. In a typical experiment [11], participants see two objects in a display, an informative spatial cue is then briefly presented at one end of an object such as a rectangle, which in turn is followed by a target. The target is presented at the cued location on most of the trials (valid trials) and at one of two uncued locations on some of the trials (the invalid trials). Importantly, the two uncued locations are equidistant from the cue, but one of them is at the other end of the cued object while the other is on a different object. Thus, on invalid trials, the cue and the target are either within the same object (the invalid same-object condition) or in two different objects (the invalid different-object condition). Responses to the target are faster on the valid than invalid trials, demonstrating space-based attentional facilitation. Responses are also faster in the invalid same-object condition compared with the invalid different-object condition, demonstrating object-based attentional selection. 

Several mechanisms have been proposed to explain this same-object advantage, or object effect (see [8] for a review). The three main ones are sensory enhancement, which emphasizes the increased sensory representation of the selected object [12,13,14,15,16,17,18,19]; attentional prioritization, which stresses the biasing of attentional scanning order that favors the selected object ([20,21]; but see [13,14]); and attentional shifting, which focuses on the additional cost in attentional shifts when attention moves from a location within an object to a location in a different object [22,23]. Despite their different emphases, it is important to note that these mechanisms are not necessarily exclusive of one another, and that more than one mechanism may contribute to object-based attention in a given paradigm. Furthermore, object-based guidance of attention is not an automatic process, but a process subject to strategic control [24,25].

Interestingly, although the object effect has been reported in many studies and is generally regarded as a robust phenomenon, the effect is less reliable in tasks that require participants to compare two simultaneously presented stimuli [23,26,27,28]. In some cases, instead of a same-object advantage, a same-object cost is found [27,29,30,31,32]. For example, in one experiment by Al-Janabi and Greenberg [29], the researchers used ovals as the objects and letters or shapes as the targets in two visual comparison tasks. The targets were presented either in the same object or in different objects. The authors combined the results from the two tasks and found a same-object advantage when the ovals were horizontally oriented. When the ovals were vertically oriented, a same-object cost was found instead. A similar object by orientation interaction was also reported in Harrison and Feldman [31], who measured sensitivity to emerging objects and object-based guidance of attention. Participants compared two features that belonged either to the same apparent object or different apparent objects. Performance was superior in the same-object condition than in the different-object condition when the objects were horizontal. When they were vertical, there was a slight two-object benefit rather than two-object cost. 

More recently, Chen and Cave [26] conducted a series of experiments that required participants to compare two letters as being the same or different. In one experiment (Experiment 1), the participants were shown two rectangles oriented either horizontally or vertically, and the letters, which were presented either simultaneously or sequentially, could appear on the same rectangle or on two different rectangles. The results showed an asymmetry in object effects, i.e., a significant same-object benefit on the horizontal rectangle trials, but a significant same-object cost on the vertical rectangle trials. Importantly, these results, which replicated the findings reported in previous research [29,33], were observed only when the data were organized by the orientation of the rectangles. Chen and Cave explained this as a result of confounding the object orientation with the target configuration orientation. This is because on horizontal rectangle trials, the targets were horizontally aligned in the same-object condition, but vertically aligned in the different-object condition; conversely, on vertical rectangle trials, the targets were vertically aligned in the same-object condition, but horizontally aligned in the different-object condition. As responding to horizontally configured stimuli that cross the vertical meridian tends to be faster than responding to vertically configured stimuli that cross the horizontal meridian [34,35], both the same-object advantage on horizontal rectangle trials and the same-object cost on vertical rectangle trials could be explained in terms of a horizontal benefit rather than object-based guidance of attention. Indeed, when the same data were organized by the orientation of the target configuration instead of the orientation of the rectangles, the object effect disappeared. In subsequent experiments, Chen and Cave showed that even when object effects were found in some conditions (e.g., by making the orientation of the rectangles predictive of the orientation of the target configuration), it was unlikely that these effects demonstrated object-based attentional guidance because the same pattern of data was found when the rectangles were removed and replaced with a salient orientation cue. These results indicate that many object effects reported in previous research that used two-stimulus comparison tasks could reflect a horizontal target configuration benefit rather than object-based attention.

Although there was no evidence of object-based attention in Chen and Cave’s [26] comparison tasks, object effects have been reported in a variety of other studies using the spatial cuing paradigm [11,14,36,37], and there is plenty of evidence that perceptual group or object boundaries can guide visual attention [2,5,38,39]. If attention can be allocated according to object boundaries, then previewing an object with both horizontal and vertical axes before the onset of the target stimuli should facilitate the allocation of attention in both directions, thereby reducing the magnitude of the horizontal benefit. We tested this hypothesis in the experiments reported here.

As it turns out, the horizontal benefit is not affected much by the presence of object boundaries in these experiments, providing additional evidence that the horizontal benefit is independent of object-based attention. These experiments also investigated the factors that would influence stimulus comparison when the orientation of the target configuration did not match the cue. In several experiments, Chen and Cave [26] found that when an informative salient orientation cue preceded the onset of the targets, response latencies to the targets changed very little regardless of the cue–target orientation congruency so long as the target configuration was horizontal. In contrast, when the target configuration was vertical, responses were substantially delayed on incongruent trials. Thus, the attentional set cost occurred only when participants were cued to expect a horizontal arrangement of the targets but then encountered a vertical arrangement. Moreover, this pattern of data emerged only when the cue was direct and informative (e.g., a pair of oriented rectangles or a large oriented bar, both with 75% validity), but not when it was direct but uninformative, or informative but symbolic (e.g., using the letter “H” and “V” to indicate orientation). These results were explained in the framework of the horizontal benefit. The attentional set does not matter very much for horizontally arranged stimuli because they are compared very quickly. The attentional set affects vertically arranged stimuli because they are processed more slowly, but setting up the correct orientation set can offset the extra time needed to process the stimuli. Furthermore, establishing an orientation set that affects performance requires both an effective stimulus-driven orientation inducer and goal-driven motivation.

A methodological feature in Chen and Cave ([26]; see Figure 6) was that the cue differed from the spatial cues used in many experiments; it was predictive of the orientation of the target configuration but not the exact locations of the targets. While the cue was always presented at fixation at the center of the display, the two targets could appear at one of four pairs of locations: they could be aligned horizontally either above or below fixation, or they could be aligned vertically either to the left or right of fixation. In other words, even when the cue was valid (e.g., a horizontally oriented bar followed by horizontally configured targets), the targets were equally likely to appear at one of two sets of locations (e.g., above or below the fixation). Thus, for the cue to be effective, it could not simply be treated as a direct spatial cue that could naturally and automatically draw attention to its location, as the targets would not appear at the location of the cue. Instead, the correct orientation set might only be established if a rather complex higher-level interpretation is applied to the cue in order to activate an orientation set that matches the cue. 

In the present study, we explored the effect of an informative image cue on the attentional set cost when the cue was predictive of both the orientation and the location of the target configuration. Comparing these results against Chen and Cave’s [26] results would test how cuing an orientation at a specific location differs from cuing an orientation independently of location. If the activation of the orientation set depends on some higher-level interpretation of the cue, switching from Chen and Cave’s orientation-only cue to an orientation-plus-location cue may weaken or eliminate the asymmetry in the attentional set cost. This is because an informative location image cue can guide attention directly to the target location, which in turn would render cue interpretation unnecessary. A long- and a short-cue condition are both included in the present experiments to explore the effect of direct location cuing.

In the two experiments reported here, participants saw an oriented configuration of two target letters preceded by an informative cue, and the task was to compare whether the targets were the same or different. The stimuli were presented either on an object or directly against a homogenous background, and the location and orientation of the cue was either congruent or incongruent with the locations and orientation of the target configuration. Experiment 1 used a mixed-design with the object factor (object-present vs. object-absent) manipulated as a between-subjects variable. Experiment 2 used a within-subjects repeated-measures design. Of particular interest was whether presenting the stimuli within an object would decrease the magnitude of the horizontal benefit and whether there would be an asymmetry in the attentional set cost between the horizontal and vertical target configuration trials.

The most important conclusion to arise from these experiments is about the two different types of attentional sets for orientation. The attentional set that arises in these experiments is a type of spatial selection and is made possible because the cue specifies location as well as orientation. This attentional set clearly differs from the orientation set in Chen and Cave [26], which was independent of location, because the attentional set in the current experiments shows no asymmetry between horizontal and vertical orientations. These new experiments also demonstrate the consistency of the horizontal advantage that was also seen in Chen and Cave. Interestingly, although this experimental paradigm originated in investigations of object-based attention, the object boundaries are notable in these experiments for what they do not do; they do not contribute to the horizontal benefit, or to the symmetric nature of the attentional set.

## 2. Experiment 1

In addition to manipulating the presence or absence of an object and the cue–target congruency in Experiment 1, we also varied the length of the cue so that it was long on half the trials, and short on the other half. In the long-cue condition (13.6° of visual angle), the ends of the cue overlapped with the locations of the subsequent targets on valid trials, and thus the presentation of the cue might draw attention more automatically to these locations [40,41]. In the short-cue condition (2.5° of visual angle), there was no overlap between the area occupied by the cue and the target locations. We had no a priori prediction about the effect of the cue type on the attentional set cost. On the one hand, unlike the cue used in Chen and Cave [26], both cues in the present experiment were predictive of target locations. If Chen and Cave’s unusual cuing results are related to the creation of an orientation set under locational uncertainty, then this experiment should not produce the same asymmetrical pattern. On the other hand, unlike the long-cue condition, the targets in the short-cue condition would not appear at the location occupied by the cue even on valid trials. Some level of cue interpretation may be necessary to set up the orientation set in anticipation of the targets, and this might produce an asymmetry in the orientation set cost in short-cue condition, but not in the long-cue condition.

## 3. Method

*Participants*. Forty undergraduate students from the University of Canterbury took part in the experiment in return for course credit. They were randomly and equally assigned to two groups. All participants gave their informed consent for inclusion before they participated in the study. The study was conducted in accordance with the Declaration of Helsinki, and the protocol was approved by the University of Canterbury Human Ethics Committee (HEC 2018/04/LR-PS).

*Apparatus and stimuli***.** Testing was carried out on two PCs, each with a 50 cm (width) × 30 cm (height) monitor. E-Prime 2.0 was used to generate stimuli and collect responses. Participants were tested individually in two dimly lit rooms at a viewing distance of approximately 60 cm. 

**Figure 1:**
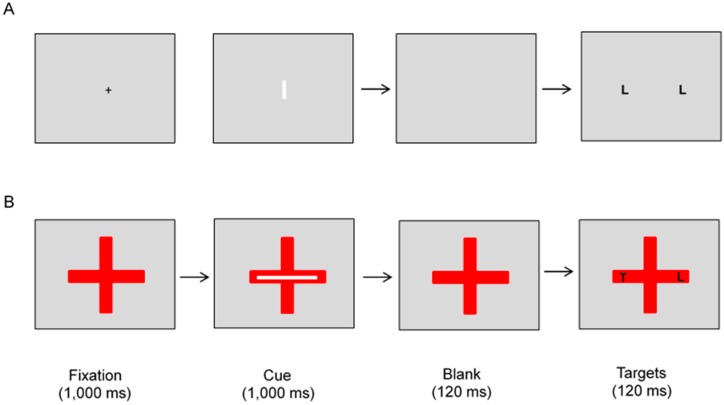
Examples of trials from Experiment 1. On each trial, participants saw a long or a short bar oriented horizontally or vertically, and this was followed by two target letters that were either horizontally or vertically aligned. The validity of the cue was 75%. For one group of participants (the object-present group), all the stimuli were shown on a red cross. For the other group of participants (the object-absent group), the stimuli were presented directly against a grey background. For all participants, the task was to judge whether the two target letters were the same or different. (**A**) An example of an object-absent, incongruent trial with a short cue. (**B**) An example of an object-present, congruent trial with a long cue. Note that the figure is not drawn to scale.

All stimuli were presented against a homogenous grey background for one group of participants (the object-absent group), but on a large red cross for the other group (the object-present group). For the object-absent group, each trial consisted of a small central fixation cross, followed by an informative cue, and then two simultaneously presented target letters (see Figure 1A). The fixation cross was black and subtended 0.3° in width and length. The cue, which could be long or short, was either a 13.6° × 0.7° or 2.5° × 0.7° elongated bar. In both conditions, it was white and presented centrally in a horizontal or vertical orientation in equal numbers of trials. The targets, which were black, were two Ts, two Ls, or one T and one L. Each letter subtended 0.67° in width and 0.76° in height. On half the trials, the letters were shown in a horizontal configuration along the horizontal meridian. On the other half, they were displayed in a vertical configuration along the vertical meridian. The letters were equally likely to be identical or different, and the center-to-center distance between the letters was 10.7°.

For the object-present group, the stimuli were the same as those described above except for two differences (see Figure 1B). First, the small black fixation cross at the beginning of a trial was replaced by a large red cross at the center of the screen. The cross was 14.2° in length along both the horizontal and vertical direction, and the width of each arm was 2.8°. Second, both the cue and the targets were presented on the red cross. The cross remained on the screen and went off together with the targets. 

*Design and procedure.* The experiment used a 2 × 2 × 2 × 2 mixed design, with object (present vs. absent) as a between-subjects factor; cue type (long vs. short), target configuration (vertical vs. horizontal), and cue–target congruency (congruent vs. incongruent) as within-subjects factors. Whereas cue type was varied across session, target configuration and cue–target congruency were both varied within a block, with different types of trials selected randomly. Half the participants were in the object-present group, while the other half were in the object-absent group. Within each group, half completed the long-cue session before the short-cue one, and this order was reversed for the other half. 

For the object-absent group, each trial began with the central fixation cross. For the object-present group, the fixation display consisted of the centrally located large red cross, and it remained on the screen until the offset of the targets. For both groups, the cue was then presented for 1000 ms. The cue was equally likely to be long or short, and equally likely to be horizontal or vertical. Upon its offset and after an interval of 120 ms, the two target letters were shown for 120 ms. The letters were horizontally aligned on half the trials, and vertically aligned on the rest of the trials. Cue validity was 75%. In other words, on three-quarters of the trials, the targets would appear at the locations indicated by the cue. On the rest of the trials, they would appear at the other locations. The intertrial interval was 500 ms. 

The task was to judge whether the two target letters were the same or different. The participants used the index and middle fingers of their right hand to press one of the two labelled keys on the number pad of a computer keyboard (the “4” key if the letters were the same and the “5” key if the letters were different). They were told to keep their eyes at the center of the screen for the duration of a trial, and both speed and accuracy were emphasized.

The experiment began with two short blocks of practice trials, with 12 trials in each block. For each participant, the order of the blocks (i.e., long-cue trials followed by short-cue ones or vice versa) followed the order of the session in the main experiment. They then completed six blocks of experimental trials, with 128 trials in each block and three blocks in each session. Participants were encouraged to take a short break after each block. The experiment took approximately 45 min to complete.

## 4. Results and Discussion

The data from two participants, both in the object-absent group, were excluded from analyses due to high error rates, which exceeded 30% in one or more conditions. For each of the remaining participants, trials above or below two standard deviations were excluded from both RT and error analyses. This resulted in the exclusion of 3.3% of the data. 

**Figure 2:**
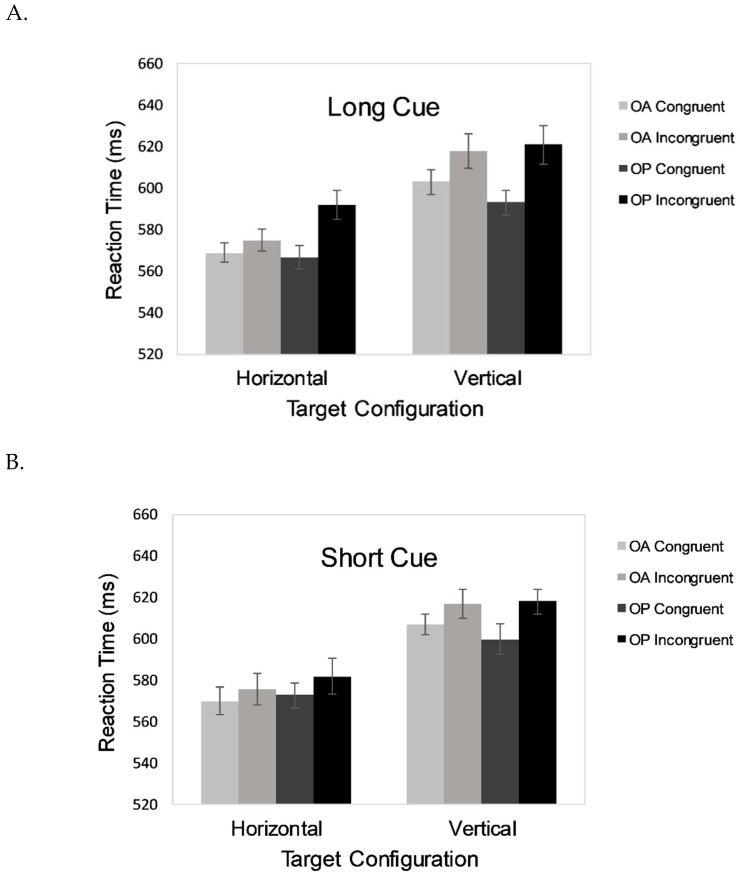
Mean Reaction time results from Experiment 1. (**A**) The long-cue condition, in which the cue was a long bar, and the subsequent targets appeared at the locations occupied by the cue on valid trials. (**B**) The short-cue condition, in which the cue was a short bar, and the subsequent targets appeared at the locations indicated, but not occupied, by the cue on valid trials. OA = object-absent trials; OP = object-present trials.

The mean RT data are shown in Figure 2A,B, and the error rates in Table 1. In all the tables and figures in this article, the error bars show the within-subjects standard error of the mean [42]. A 2 × 2 × 2 × 2 mixed ANOVA on RTs showed a significant main effect of target configuration, *F*(1,36) = 75.74, *MS_e_* = 1169, *p* < 0.001, *η*_p_^2^ = 0.68, indicating faster responses when the targets were horizontally aligned (576 ms) compared with when they were vertically aligned (610 ms). Responses were also faster in the congruent condition (585 ms) than in the incongruent condition (600 ms), *F*(1,36) = 30.20, *MS_e_* = 525, *p* < 0.001, *η*_p_^2^ = 0.46. Cue type interacted with congruency, *F*(1,36) = 7.11, *MS_e_* = 178, *p* = 0.01, *η*_p_^2^ = 0.17, indicating a larger congruency effect when the cue was long (19 ms) compared with when it was short (10 ms). Object also interacted with congruency, *F*(1,36) = 4.35, *MS_e_* = 525, *p* = 0.04, *η*_p_^2^ = 0.11, with the congruency effect being larger in the object-present group (20 ms) than in the object-absent group (9 ms). No significant interaction was found between target configuration and object, *F*(1,36) = 1.23, *MS_e_* = 1169, *p* = 0.28, *η*_p_^2^ = 0.03. This result showed that the magnitude of the horizontal benefit was not influenced by presenting the cue and the targets on an object. There was also no significant interaction between target configuration and congruency, *F*(1,36) = 2.26, *MS_e_* = 339, *p* = 0.14, *η*_p_^2^ = 0.06, indicating no evidence of an asymmetry in the attentional set cost between the horizontal and vertical trials. Finally, the interaction between target configuration and cue type was negligible, *F*(1, 36) < 1 *ns*, suggesting that cue type had little effect on the horizontal benefit. No other effects reached significance. Detailed information on the results of the statistical analyses can be found in Table A1 and Table A2 in the Appendix A. 

A similar analysis was performed on the accuracy data. Only the main effect of target configuration was significant, *F*(1,36) = 6.16, *MS_e_* = 13, *p* = 0.02, *η*_p_^2^ = 0.15. Consistent with the RT results, accuracy was higher when the targets were horizontally configured (4.1% error rate) rather than vertically configured (5.1% error rate). There was no indication of any speed–accuracy trade-offs.

As in previous research [26,34,35], we found a reliable horizontal benefit. Responses to the targets were both faster and more accurate when the targets were configured horizontally rather than vertically. It has been proposed that this effect may arise due to the differences in the efficiency of processing stimuli that cross the vertical vs. the horizontal meridian [34,35] and/or the ease at which symmetry is detected when stimuli cross the vertical compared with the horizontal meridian [27,31]. We will discuss possible accounts for this effect in more detail in the general discussion section. 

To our surprise, we found no evidence that object-based guidance of attention affected the horizontal benefit. Whether the cue and the targets were shown on an object or directly against a homogenous background did not appear to influence the magnitude of the horizontal benefit in a meaningful way. There was a small numerical reduction of the effect in the object-present group (30 ms) compared with the object-absent group (39 ms), but the effect was small (*η*_p_^2^ = 0.03) and not reliable (*p* = 0.28). We had hypothesized that letting the participants have a good preview of an object that had a salient horizontal and vertical axis would facilitate the allocation of attention in both directions. When the cue and the targets were then presented on the object, because attention had already been allocated inside the entire object, this would lead to a decrease in the horizontal benefit. However, such an effect was not found, even though there was a numerical trend in the predicted direction. 

Interestingly, presenting the stimuli on the object affected the congruency effect. Compared to the object-absent group, the participants in the object-present group showed a larger congruency effect. This effect may be caused by the physical properties of the stimuli rather than object-based guidance of attention. A white cue against a red cross is more salient than a white cue against a grey background. The increased salience of the cue in the object trials could make the orientation of the cue more pronounced, which in turn could delay the processing of the targets when the orientation of the target configuration did not match the orientation of the cue. Consistent with this line of reasoning is the finding that the increase in the congruency effect was comparable regardless of the orientation of the target configuration.

As the targets were presented against a red background in the object-present trials but against a gray background in the object-absent trials, the two types of trials differed in luminance and color contrast between the targets and the background. Is it possible that these differences also contributed to the larger congruency effect in the object-present trials? A close examination of the results revealed that this possibility is very small. If these differences had a major impact in the processing efficiency of the targets, there would be a main effect of object in RT and/or accuracy. However, there was very little difference in the overall response latencies (a difference of 1 ms, *p* = 0.96) or in the error rates (a difference of 1.2%, *p* = 0.24) between the two types of trials.

One of the goals of Experiment 1 was to identify the factors important in establishing the attentional set for orientation that was demonstrated by Chen and Cave [26]. In their experiments, the cue was predictive of the orientation of the target configuration but not the locations of the targets, and an attentional set cost was found in the vertical, but not the horizontal target configuration trials. In the present experiment, the cue was predictive of both the location and the orientation of the target configuration, and significant congruency effects of similar magnitude were found when the target configuration was horizontal (12 ms) and when it was vertical (18 ms). Furthermore, these effects did not differ between the long- and short-cue trials, indicating that the level of cue interpretation did not affect the attentional set here. The direct image cue allows the participants to prepare effectively for the target configuration that matches the cue in both orientation and location. 

To get a clearer picture of the effect of location information on the horizontal benefit, we compared the data from Experiment 4 in Chen and Cave [26] and the data from the long-cue condition of Experiment 1 in the present study. The same cue (both its size and location) was used in both experiments, but only the cue in the present study indicated the locations of the targets directly on valid trials. A 2 × 2 × 2 mixed ANOVA was conducted, with experiment as a between-subjects factor, letter configuration and cue–target congruency as within-subjects factors. For the sake of brevity, we report only the results involving experiment. There was a significant 2-way interaction between experiment and target configuration, *F*(1,34) = 7.17, *MS_e_* = 478, *p* = 0.01, *η*_p_^2^ = 0.17, with a larger horizontal benefit in Experiment 1 of the present study (38 ms) compared with Experiment 4 in Chen and Cave (19 ms). This result indicates that location uncertainty reduces the magnitude of the horizontal benefit. There was also a marginally significant 3-way interaction of target configuration, congruency, and experiment, *F*(1,34) = 3.74, *MS_e_* = 187, *p* = 0.06, *η*_p_^2^ = 0.10, showing a smaller difference in the congruency effects between the horizontal and vertical target configuration trials in the present experiment (a difference of 9 ms) compared with Experiment 4 in Chen and Cave (a difference of 27 ms). Thus, when the cue predicts both orientation and location, there was little asymmetry in the attentional set cost between the horizontal and vertical target configuration trials. 

## 5. Experiment 2

In Experiment 1, the participants in the object-present group showed a stronger congruency effect than their counterparts in the object-absent group, but the two groups did not differ in the magnitude of the horizontal benefit. As different groups were used in Experiment 1, there might be differences between the groups that had contributed to the observed results. To minimize this possibility, we conducted Experiment 2, in which the object was varied as a within-subjects rather than a between-subjects factor. 

## 6. Method

The method was the same in Experiment 2 as in Experiment 1 except for the following differences. First, only the long-cue trials were included in the experiment. Second, object was varied as a within-subjects factor. Thus, the design was a 2 × 2 × 2 repeated-measures design, with object (present vs. absent), letter orientation (vertical vs. horizontal), and cue–target congruency (congruent vs. incongruent) as the key manipulations. Object was a blocked manipulation with participants completing one session of object-present trials and one session of object-absent trials. Twenty-five new participants from the same participant pool took part in the experiment. Thirteen of them completed the object-present session before the object-absent session, and this order was reversed for the rest of them. 

## 7. Results and Discussion

Two participants’ data were excluded from analyses, one due to high error rates (over 20% in multiple conditions), and the other due to high RT (mean RT over three standard deviations above the mean RT for the rest of the participants). For the remaining participants, the data were treated in the same way as that in Experiment 1, i.e., trials above or below two standard deviations were excluded from both RT and error analyses. This resulted in the exclusion of 3.4% of the data. 

**Figure 3:**
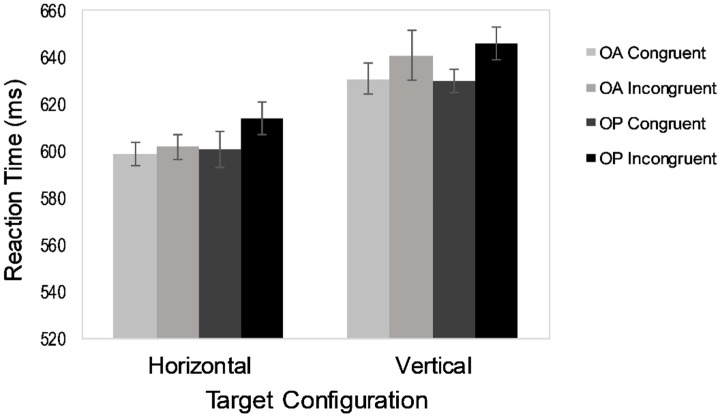
Mean reaction time results from Experiment 2. OA = object-absent trials; OP = object-present trials.

Figure 3 shows the mean RTs and Table 2 shows the error rates. A 2 × 2 × 2 repeated-measures ANOVA was conducted on the RT data. Once again, there was a robust target configuration effect, *F*(1,22) = 68.55, *MS_e_* = 1001, *p* < 0.001, *η*_p_^2^ = 0.76, indicating faster responses to horizontally configured targets (614 ms) than to vertically configured ones (653 ms). The congruency effect was also reliable, *F*(1,22) = 5.63, *MS_e_* = 810, *p* = 0.03, *η*_p_^2^ = 0.20. Participants were faster in the congruent condition (629 ms) than in the incongruent condition (639 ms). As in Experiment 1, congruency interacted with object, *F*(1,22) = 4.65, *MS_e_* = 391, *p* = 0.04, *η*_p_^2^ = 0.17, indicating a larger congruency effect in the object-present condition (14 ms) than in the object-absent group (4 ms). No significant interactions were found between target configuration and object, *F*(1,22) < 1 *ns*, or between target configuration and congruency, *F*(1, 22) < 1 *ns*. No other effects reached significance, either. 

A 2 × 2 × 2 repeated-measures ANOVA was also performed on the accuracy data. There was a marginally significant target configuration effect, *F*(1,22) = 3.65, *MS_e_* = 9, *p* = 0.07, *η*_p_^2^ = 0.14, indicating numerically higher accuracy when the targets were horizontally configured (4.1% error) rather than vertically configured (5.0% error). No other effects were significant, and there was no indication of speed–accuracy trade-offs.

The results of Experiment 2 were very similar to those of Experiment 1. In both experiments, participants showed a significant horizontal benefit and a significant congruency effect. More importantly, presenting the cue and the targets on an object again increased the congruency effect, but had no effect on the magnitude of the horizontal benefit. These results indicate that the pattern of the data observed in Experiment 1 was not due to the manipulation of object as a between-subjects factor.

As in Experiment 1, there was no evidence of an asymmetry in the attentional set cost between the horizontal and vertical trials. Although the congruency effect was numerically larger when the targets were configured vertically (12 ms) rather than horizontally (8 ms), this effect was very small (*η*_p_^2^ = 0.02). Thus, making the cue predictive of location in addition to orientation eliminated the asymmetry in the attentional set cost found in Chen and Cave [26]. 

## 8. General Discussion

Previous research using an informative orientation cue has shown that performance is better in visual comparison tasks when the targets are configured horizontally rather than vertically. Furthermore, when Chen and Cave [26] cued the orientation of an upcoming target configuration without cuing its location, there was an attentional set cost on vertical trials when the orientation of the target configuration did not match expectation. The current study replicated the horizontal benefit while also testing whether the asymmetric cuing effect arises with other types of cues, or whether it only arises when the viewer is set for a specific orientation without a set for location. In the present study, we used a cue predictive of both the orientation of the target configuration and the locations of the targets, and we explored the factors affecting the efficiency of visual comparison. Specifically, we investigated how performance was affected by the orientation of the target configuration, the presence of object boundaries, and the overlap between cue and target locations. 

***Orientation Set Cost.*** Unlike the results in Chen and Cave [26], in which an attentional set cost was found only in the vertical trials, the cue–target congruency effect in the present study was comparable regardless of the orientation of the target configuration. This result demonstrates that cuing an orientation at a specific location has a very different effect from cuing an orientation independently of location. One potentially important factor here is the informativeness and salience of the cue. In the present study, the cue was predictive of location on 75% of the trials, so participants have a strong incentive to allow attention to be allocated in response to the cue. As the targets would appear at the cued location on valid trials, there would be no need for complex high-level preparation before the cue appears, and no need for complex high-level interpretation of the cue, because the onset of the cue would attract attention to the location of the cue in a fairly automatic fashion. 

When a cue predicts only the orientation of the target configuration as in Chen and Cave [26], it may be possible to create an appropriate orientation set with a wide attentional zoom. Because the targets never appear at the actual location of the cue, if spatial attention is going to be used to facilitate response, it would need to be allocated in such a way as to include all potential target locations; in other words, it would be beneficial to disengage attention from the location of the cue while maintaining its orientation as soon as the cue is interpreted. The disengagement of spatial attention from the location of the cue may have played an important role in the results of Chen and Cave. Alternatively, the orientation set in Chen and Cave may have been achieved with a nonspatial form of attentional selection that prepares for an orientation without selecting any specific location.

It is possible that a fast change from a vertical orientation set to horizontally configured targets can only be made without much cost when attention is not glued to a specific location. When attention is focused on the wrong location, there will be a performance cost, either because attention must be disengaged from an old location before re-engaging to a new location, or because processing must proceed with a suboptimal attentional allocation. There may be a set amount of cost regardless of whether the attentional set has to change from horizontal to vertical or from vertical to horizontal. This can explain why no asymmetry in attentional set cost was found in the present study.

Comparing these results to those of Chen and Cave [26] highlights the difference between the location-specific orientation set seen here and the location-independent orientation set in the earlier study. An idea of an attentional set for a location is easy to reconcile with our visual experience; people generally find it easy to accept the notion of an attentional spotlight that selects a specific location, and we are often aware that we are tuned to receive stimuli from that location. However, the idea of an attentional set that favors a specific orientation independently of location does not fit as well with our visual experience. Not only are we generally unaware of how this orientation selection contributes to our visual processing, but it is difficult to imagine why it only comes into play with vertical stimuli. The fact that this orientation set can shape our perception in such an unusual way without our awareness makes it worthy of further investigation.

***Horizontal Benefit.*** In testing the horizontal benefit, the results were consistent across both experiments: participants showed a robust horizontal benefit. There was no evidence that the magnitude of the effect was modulated by object-based attention, or the validity or type of cues. These results confirmed and expanded what was previously known about the horizontal benefit.

In general, the studies that show the horizontal benefit, which has also been called the different-hemifield advantage [35] and horizontal–vertical anisotropy [34], share two common features: attention is paid to both sides of the meridian on a given trial and attention includes regions that are far from one another. This can be seen in the present experiments, in which two simultaneously presented targets appeared on different sides of the meridian, and the separation of the targets was about 10°. We found a robust horizontal benefit. Similar methodological features are also present in Chen and Cave [26], Davis and Holmes [27], and in Sereno and Kosslyn [35], all of which showed horizontal benefits. Barnas and Greenberg [34] used a detection task in a spatial cuing paradigm. On invalid trials, the cue and the target, whose separation was about 10°, were on different sides of the meridian, and the horizontal benefit was found. In all these studies, attention was directed or shifted across the horizontal or vertical meridian, and the zoom of attentional focus was quite broad. 

What caused the horizontal benefit? Several accounts have been proposed. Sereno and Kosslyn [35] noted that each hemisphere has its own attentional resource and each can process information independently [43,44]; in addition, stimuli within the same hemisphere interfere with one another more than stimuli across different hemispheres [45]. Thus, compared to the vertically configured stimuli that cross the horizontal meridian (i.e., within-hemisphere stimuli), the horizontally configured targets that cross the vertical meridian (i.e., between-hemisphere stimuli) have an advantage. This advantage can be due to the between-hemisphere stimuli having additional resources that facilitates processing, or the within-hemisphere stimuli having greater competition that delays processing, or both. These hemisphere-specific mechanisms can explain the horizontal benefit observed in the present study. Barnas and Greenberg [34] explained their results in a similar way. 

The horizontal benefit can also be explained in terms of symmetry detection. Based on previous research that shows faster symmetry detection when the axis is oriented vertically than horizontally ([46,47], and see [48] for a review), both Davis and Holmes [27] and Harrison and Feldman [31], who used same/different comparison tasks in their studies, pointed out that the difference in processing efficiency in symmetry detection associated with vertical vs. horizontal axes can explain why performance is better for horizontally arranged stimuli that cross the vertical meridian than for vertically arranged stimuli that cross the horizontal meridian. However, it should be noted that although the symmetry account can explain the horizontal benefit in studies that use same/different comparison tasks (e.g., the present experiment), it is more difficult to explain the effect in studies that do not involve comparison, such as when the task is to detect the presence of a pre-defined target [34]. 

To our surprise, viewing an object (i.e., a red cross) that has equally salient horizontal and vertical axes before responding to stimuli presented on that object did not reduce the horizontal benefit. This result indicates that the horizontal benefit may be driven primarily by the stimulus configuration and its orientation, with little influence from object-based guidance of attention, at least in the present paradigm. Although the absence of the object effect was somewhat unexpected, this finding is nevertheless in general agreement with the accounts that have been proposed to explain the horizontal benefit, which emphasize the role of hemispheric-specific processes [34,35] or symmetry detection mechanisms [27,31]. In the present experiments, it is likely that while attention was allocated to the entire object (i.e., the red cross) when it was in view, the onset of the cue, which remained on the screen for a full second, re-directed attention to the cue, and this eliminated any object-based effect on the subsequent task.

The consistency of the horizontal benefit across different conditions suggests that it might be explained by a low-level mechanism that is not associated with attention or object representations. One well established low-level effect that is relevant for these stimuli is the horizontal–vertical anisotropy, with greater contrast sensitivity and faster processing speed when stimuli are on the horizontal compared with the vertical midline [49,50]. Abrams et al. proposed that these performance asymmetries are best explained in terms of the anatomical organization of the early vision system, which has higher cone density in the retina and larger space devoted to the representation of stimuli in V1 along the horizontal meridian than the vertical meridian [51,52,53]. Thus, even though the letters to be compared in our experiments all appear at the same distance from fixation, we can expect the letters in the horizontal condition to benefit from higher sensitivity than those in the vertical condition, which might help to explain the horizontal benefit seen here. However, the asymmetry is weaker for locations farther from the midlines, and so the midline asymmetry account does not fit as well with the horizontal benefit demonstrated in Chen and Cave [26], in which the target letters were never presented on the horizontal or the vertical meridian, and each letter is equally far from the horizontal and vertical midlines, and thus each letter would be equally affected by the horizontal/vertical asymmetry. There is also an asymmetry that favors the lower visual field compared to the upper visual field to consider here [49]. In Chen and Cave’s stimulus configuration, the horizontal letter pairs in the lower field may be perceived better than those in the upper field. In their vertical condition, each letter pair to be compared will have one high-sensitivity lower letter and one low-sensitivity upper letter. In both conditions, any upper/lower field differences would probably be averaged out, making it unlikely that Chen and Cave’s horizontal advantage could result from these asymmetries. Thus, at least part of the horizontal advantage seen in these new experiments is also probably not attributable to these asymmetries.

***Object Effects.*** Interestingly, presenting the stimuli on an object increased the congruency effect compared with presenting the stimuli directly on a homogenous background. One possible explanation of this effect is the increased salience of the cue in the object-present condition (a white cue against a red background) compared with the object-absent condition (a white cue against a grey background). As we mentioned in the discussion of Experiment 1, the increased salience of the cue in the object-present condition could strengthen the representation of the cue orientation, which in turn could delay the processing of the targets when the orientation/location of the target configuration did not match that of the cue. This interpretation is also consistent with the results of Experiment 2. The object by congruency interaction was driven primarily by an increase in the cost of being invalidly cued in the object condition compared with the no-object condition. There was little difference in the congruency trials between the two conditions.

There is another possibility for the increased congruency effect in the object-present condition. If the speed of attentional movement is influenced by the complexity of the region between the movement initiation point and the movement end point, then response latencies should be longer on invalid trials in the object-present condition than in the object-absent condition. This is because while attention only needed to move across a single homogenous region in the object-absent condition (i.e., the grey background), it had to move across two different regions with different depth (i.e., the red cross and the grey background) in the object-present condition. It is likely that both salience and complexity played a role in the results found in the present study. 

In summary, the present study shows that cuing the location of the target configuration eliminated the asymmetry in the attentional set cost found in previous research [26]. This difference illustrates that there are two different ways in which attention can use orientation information to prepare for an upcoming stimulus. Furthermore, these experiments provide new evidence that visual comparison is more efficient when the target configuration is horizontal rather than vertical, and that this horizontal benefit is not modulated by object-based attention. These results add to a growing body of research that demonstrates differential processing efficiency as a function of target configuration. They also underscore the necessity for theories of attention to take into account the orientation of attentional distribution as an important factor in modulating the efficiency of visual information processing. 

## Figures and Tables

**Table 1:** Mean error rates (percent incorrect) as a function of object, cue type, target configuration orientation, and cue-target congruency in Experiment 1. Within-subject standard errors are in the parentheses.

**Object-Absent Group**				
**Cue Type**	**Horizontal**		**Vertical**	
	**Congruent**	**Incongruent**	**Congruent**	**Incongruent**
Long	3.7 (0.5)	2.9 (0.5)	4.8 (0.6)	4.1 (0.5)
Short	3.7 (0.5)	2.7 (0.7)	4.5 (0.6)	5.2 (0.8)
**Object-Present Group**				
**Cue Type**	**Horizontal**		**Vertical**	
	**Congruent**	**Incongruent**	**Congruent**	**Incongruent**
Long	4.7 (0.6)	5.1 (0.7)	5.4 (0.5)	5.4 (0.6)
Short	5.0 (0.6)	4.6 (0.5)	5.7 (0.6)	5.3 (0.7)

**Table 2:** Mean error rates (percent incorrect) as a function of object, target configuration orientation, and cue-target congruency in Experiment 2. Within-subject standard errors are in the parentheses.

Object	Horizontal		Vertical	
	Congruent	Incongruent	Congruent	Incongruent
Absent	4.4 (0.4)	4.3 (0.6)	4.9 (0.5)	5.2 (0.6)
Present	4.0 (0.5)	3.8 (0.5)	5.0 (0.5)	4.9 (0.6)

## Appendix A

**Table A1:** Results of ANOVAs on the reaction times and error rates in Experiment 1 as a function of object, cue type, target configuration, and cue-target congruency.

	Reaction Times			Error Rates		
	*F* (1,36)	*p*	η_p_^2^	*F* (1,36)	*p*	η_p_^2^
Obj	<0.01	0.96	<0.01	1.45	0.24	0.04
Cue	0.01	0.91	<0.01	0.04	0.83	<0.01
Cue*Obj	<0.01	0.94	<0.01	0.05	0.82	<0.01
TConf	75.74	<0.001	0.68	6.16	0.02	0.15
TConf*Obj	1.22	0.28	0.03	0.85	0.36	0.02
Cong	30.20	<0.001	0.46	0.73	0.40	0.02
Cong*Obj	4.35	0.04	0.11	0.34	0.56	<0.01
Cue*TConf	0.37	0.55	0.01	0.30	0.59	<0.01
Cue*TConf*Obj	0.22	0.64	<0.01	0.06	0.81	<0.01
Cue*Cong	7.11	0.01	0.17	<0.01	0.96	<0.01
Cue*Cong*Obj	2.94	0.09	0.08	1.00	0.32	0.03
TConf*Cong	2.26	0.14	0.06	0.44	0.51	0.01
TConf*Cong*Obj	<0.01	0.95	<0.01	1.38	0.25	0.04
Cue*TConf*Cong	<0.01	0.99	<0.01	0.79	0.38	0.02
Cue*TConf*Cong*Obj	0.80	0.38	0.02	0.32	0.58	<0.01

Note: Obj = Object; Cue = Cue type; TConf = Target configuration; Cong = Cue-target congruency.

**Table A2:** Results of ANOVAs on the reaction times and error rates in Experiment 2 as a function of object, target configuration, and cue-target congruency.

	Reaction Times			Error Rates		
	*F* (1,22)	*p*	η_p_^2^	*F* (1,22)	*p*	**η_p_^2^**
Obj	0.57	0.46	0.03	0.29	0.59	0.01
TConf	68.55	<0.001	0.76	3.64	0.07	0.14
Cong	5.63	0.03	0.20	<0.01	0.96	<0.01
Obj*TConf	0.34	0.57	0.02	0.17	0.69	<0.01
Obj*Cong	4.65	0.04	0.17	0.23	0.63	0.01
TConf*Cong	0.51	0.48	0.02	0.14	0.71	<0.01
Obj*TConf*Cong	0.18	0.67	<0.01	0.01	0.91	<0.01

Note: Obj = Object; TConf = Target configuration; Cong = Cue-target congruency.
